# More than meets the AI: The possibilities and limits of machine learning in olfaction

**DOI:** 10.3389/fnins.2022.981294

**Published:** 2022-09-01

**Authors:** Ann-Sophie Barwich, Elisabeth A. Lloyd

**Affiliations:** ^1^Department of History and Philosophy of Science and Medicine, College of Arts and Sciences, Indiana University Bloomington, Bloomington, IN, United States; ^2^Cognitive Science Program, College of Arts and Sciences, Indiana University, Bloomington, IN, United States

**Keywords:** philosophy of science, neurobiology, mechanisms, stimulus response, structure odor relationship, medicinal chemistry, logic of research questions, receptor modeling

## Abstract

Can machine learning crack the code in the nose? Over the past decade, studies tried to solve the relation between chemical structure and sensory quality with Big Data. These studies advanced computational models of the olfactory stimulus, utilizing artificial intelligence to mine for clear correlations between chemistry and psychophysics. Computational perspectives promised to solve the mystery of olfaction with more data and better data processing tools. None of them succeeded, however, and it matters as to why this is the case. This article argues that we should be deeply skeptical about the trend to black-box the sensory system’s biology in our theories of perception. Instead, we need to ground both stimulus models and psychophysical data on real causal-mechanistic explanations of the olfactory system. The central question is: Would knowledge of biology lead to a better understanding of the stimulus in odor coding than the one utilized in current machine learning models? That is indeed the case. Recent studies about receptor behavior have revealed that the olfactory system operates by principles not captured in current stimulus-response models. This may require a fundamental revision of computational approaches to olfaction, including its psychological effects. To analyze the different research programs in olfaction, we draw on Lloyd’s “Logic of Research Questions,” a philosophical framework which assists scientists in explicating the reasoning, conceptual commitments, and problems of a modeling approach in question.

## The scientific challenge: Decoding the nose

**FIGURE 1:**
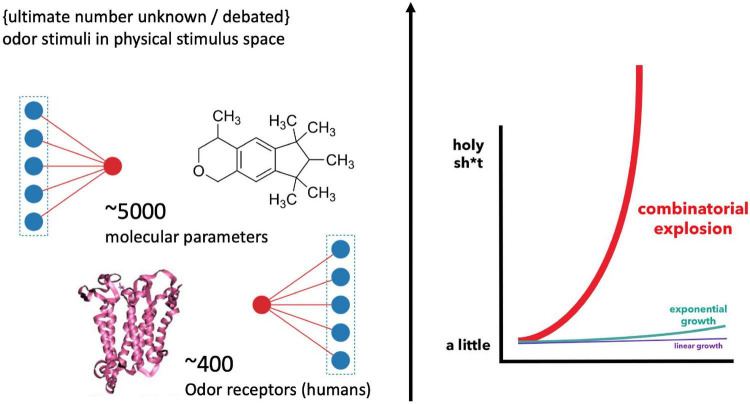
Illustration of the “big data” challenge in modeling olfaction [Graph, right, by Bair (2015)]. Humans feature about 400 receptor genes. Each receptor interacts combinatorically with multiple physico-chemical properties of multiple odorants (Malnic et al., 1999) [The precise number of possible stimuli detected by the olfactory system is unknown and debated (Meister, 2015); while it is considered high (Ohloff et al., 2012)]. Additionally, there are several thousand possible parameters involved in odorant-receptor binding, resulting in an explosion of possible combinations for stimulus-receptor interactions.

How odor quality is encoded in molecules remains an enigma in modern neuroscience. In 1991, when Buck and Axel (1991) discovered the odor receptor genes belonging to the superfamily of GPCRs (G-protein coupled receptors), hopes were raised that this molecular riddle could be solved soon. Yet, the opposite has been the case. The reason for this absence of a conclusive scientific classification and prediction of sensory qualities in chemical compounds is not for a lack of experimental progress. Instead, 30 years later, and the olfactory pathway has revealed a complexity that added several more conditions and layers to its information processing. The number of possible combinations by which chemical features are encoded by the olfactory receptor cells are mind-boggling (Figure 1), explaining why “[t]his stimulus-percept problem has been difficult to solve in olfaction,” especially “because odors *do not vary continuously* in stimulus space,” while “the size and dimensionality of olfactory perceptual space is unknown” (Keller et al., 2017; added emphasis). This suggest that there might not be a clear-cut correspondence in properties and structure between models of the physical stimulus and perceptual space.

Advances in new computational tools are often portrayed as a promising avenue through this complex molecular forest *en route* to a comprehensive stimulus-response model. Models of the chemical stimulus in olfaction serve a critical explanatory function: “Understanding the relationship between a stimulus and how it is perceived reveals fundamental principles about the mechanisms of sensory perception” (Keller and Vosshall, 2016). Knowing what kinds of molecular features are causally responsible for specific perceptual effects may facilitate better insight into the biological processes that bring about these effects, or so it is assumed. Machine learning models in olfaction hold the promise of obtaining such generalizable structure-odor rules that could reduce the vast chemical complexity of the stimulus. While interest in these models is increasing, we point out a central oversight in their current design and application.

This article discusses the danger of the hidden assumptions present in current applications of machine learning (ML in the following) in olfaction. Our analysis extends previous critiques of ML-models in biological systems that cautioned of methodological pitfalls, including incomplete data and small datasets or false positives, opacity in algorithm design, and a lack of empirical and contextual grounding (Johnson, 2008; Coveney et al., 2016; Ratti and López-Rubio, 2018; London, 2019; Ratti and Graves, 2022). The scientifically important issue raised with this analysis is that “[these pitfalls do] not explain *how we arrive* at the wrong model, just *how we accept* the wrong model” (Johnson, 2008, 25, added emphasis).

Our article makes the following claim: current computational approaches to modeling the olfactory stimulus that are based on the principles of analytic chemistry target the wrong causal features of the stimulus. These approaches provide mistaken answers to research questions investigating the molecular features as the cause of odor perception. Specifically, they ask the wrong research *questions*, given twenty-first century discoveries about the neurobiology of smell, and therefore end up with the wrong answers. Instead, we argue that stimulus models ought to be based in biology, particularly in receptor responses to the chemical stimulus of smell. We demonstrate that stimulus models based in chemistry and biology are not co-extensive, meaning they need not identify the same chemical features as causally responsible for olfactory signaling and odor quality.

Against our claim stands a key conviction held by many ML-proponents, who characterize computational tools as theory-neutral and share the assumption that successful algorithms identify the causally responsible features—independently of empirical insights into receptor behavior and the biology of the system. This view is visible in big money projects such as Google AI which joined the race to crack the code in the nose. A member of the Google AI team, Wiltschko (2019; added emphasis), confidently proclaimed: “*Based on analogous advances in deep learning for sight and sound*, it should be possible to *directly predict the end sensory result of an input molecule*, even *without knowing the intricate details of all the systems involved*.” This view is shockingly uninformed, starting with the assumption that olfaction works analogously to vision and audition, which is simply not the case (Barwich, 2019, 2020a). A critical difference is the vast genetic diversity of the olfactory system compared to vision and audition. The challenge of modeling the stimulus space in olfaction thus goes beyond chemical complexity. It concerns high stimulus-response variation based on a genetically highly heterogeneous sensory system, resulting in divergent perceptual responses to physico-chemical information (Barwich, 2020a,forthcoming). Thus, our paper centers on the second premise expressed by Wiltschko and others: that one can eschew insights from the biology of the system one wants to model in favor of an algorithm that magically extracts just the right features.^1^

This is both *logically unjustified* and *empirically wrong*. We support our claim by extracting the logic and framing of, and then contrasting, the experimental designs and results of the two most successful contenders for each modeling approach. On the one hand, we look at Keller et al.’s (2017) *Science* paper that used ML-algorithms to predict odor quality from the chemical structure of odorants. On the other hand, we analyze Poivet et al.’s (2016, 2018) wet-lab studies examining the responses of odor receptors to selected odorants to identify which chemical features of these odorants are causally responsible. For comparison of these two approaches, we draw on Lloyd’s (2015) “Logic of Research Questions” (LRQ), a philosophical framework which assists scientists in explicating the reasoning, conceptual commitments, and problems of a modeling approach in question.

Research programs are compared in terms of the logic of their implicit research questions including their possible and responsive answers, and the standards of evidence required for these answers to be met. Our analysis using LRQ presents two heavy blows to optimism about current ML-approaches in olfaction. First, stimulus models based on receptor biology yield crucial differences in their selection and hierarchy of causally responsible molecular features compared to stimulus models based in traditional analytic chemistry. Second, studies in receptor biology revealed molecular features as critical to odor detection that were not identified in previous ML-models. Consequently, this article emphasizes, for ML-techniques to truly advance understanding of olfaction and its stimulus, they must be modeled after the characteristics of the system it is consulted to assist in modeling, instead of black-boxing its causality.

We proceed as follows. Section “Deep nose and the logic of research questions: theory in machine learning studies in olfaction” looks at the current promises of ML-models in olfaction and then introduces the Logic of Research Questions, a philosophical framework that helps to carve out the conceptual differences between chemistry-based and biology-based models of the stimulus. Section “Analysis: chemistry-based versus receptor-response models” evaluates the available empirical evidence of the leading studies for each approach in support of our analysis. Here we will see how receptor-response studies correct chemistry-based modeling and widen our explanatory possibilities on odor coding. Section “Conclusion” presents our conclusions.

## Deep nose and the logic of research questions: Theory in machine learning studies in olfaction

Computational techniques are expected by advocates of the no-theory ML-paradigm to systematically link molecular features with olfactory quality to arrive at a model matching the physical stimulus space with perceptual quality space. Most of these studies (e.g., Koulakov et al., 2011; Kumar et al., 2015; Kepple and Koulakov, 2017; Gutiérrez et al., 2018) compare two kinds of datasets: verbal descriptors (e.g., “orange,” “garlicky”) and molecular parameters (e.g., benzene rings, number of carbon atoms, molecular weight, etc.). Despite an extensive research tradition mining for correlations between these datasets (Rossiter, 1996; Sell, 2006), to this day, these tools have not found sufficient success in facilitating generalizable “structure-odor rules” (SORs) that would allow predicting the odor of a molecule from its molecular composition. One explanation for this shortcoming, according to its proponents, is the quality and composition of the datasets, as “many [of these studies] relied on psychophysical data from a single 30-year-old [trade lexicon] that used odorants with limited structural and perceptual diversity” (Keller et al., 2017).

A ML-study, published in *Science* and confidently entitled “Predicting human olfactory perception from chemical features of odor molecules” (Keller et al., 2017), aimed to correct this flaw by drawing on a newly collected psychophysical dataset (Keller and Vosshall, 2016). This study set out to provide a proof of principle and might be considered the leading-edge work of this kind today. It received positive attention from science writers (Yong, 2016) with its declaration that it is “possible to predict the perceptual qualities of *virtually any molecule* with an *impressive degree of accuracy* to reverse-engineer the smell of a molecule” (Keller et al., 2017; added emphasis).

We advise caution concerning this conclusion and contend this claim. But we want to emphasize right up front that our central point of disagreement is not aimed at the consultation of novel powerful technologies for solving older empirical problems, or the auspicious tool of ML as such. On the contrary, ML has proven indispensable for the toolbox of science and even superseded expectations in some areas of biology, most remarkably in modeling protein folding (e.g., *AlphaFold*, Jumper et al., 2021). Experimental and theoretical breakthroughs throughout the history of (neuro)science fundamentally hinge on the development and application of new tools (Churchland, 1995; Gigerenzer and Goldstein, 1996; Schickore, 2007; Bickle et al., 2021). Rather, our critique targets the theoretical and empirical *adequacy of the assumptions hidden in the current use* of ML-models in olfaction.

The attraction of ML lies in its potential to provide shortcuts for the evaluation of otherwise complex and impenetrable data correlations. ML-techniques *seem* to espouse a theory-neutral approach, or something close to theory-neutral modeling, in that they do not directly rely on researchers’ intuitions about what the data might mean as these tools “merely” mine the data for salient and systematic correlations hidden by complexity. Therefore, the true concern for big data advocates is not the making of inferences from data mining operations *per se*, but the quality and size of datasets used.

The case of olfaction proves this optimism misplaced. Neither is ML theory-neutral nor are its correlations explanatory by their own merit. What makes data correlations potentially explanatory is the causal model to which ML-correlations are applied. Concern about the use of ML-tools in olfaction aligns with broader criticisms regarding the rise of big data and its associated techniques:

Not only do these methods invariably require far larger quantities of data than anticipated by big data aficionados in order to produce statistically reliable results, but they can also fail in circumstances beyond the range of the data used to train them because they are not designed to model the structural characteristics of the underlying system (Coveney et al., 2016).

But are the comparatively small public^2^ datasets in olfactory psychophysics the reason for a lack of success in current ML-models of SORs? One may suggest that an AI worth its hype *should* be able to solve even a smaller and messy dataset (especially considering receptor studies yielding highly revealing results despite using a small number of compounds; see section “Biology makes scents of chemistry: receptor-response models of the stimulus”). Thus, we want to focus on the second point of criticism: if ML-models in olfaction continue to “black-box” the structural conditions and biological mechanisms of the underlying system, they inevitably result in contrived and potentially misleading correlations.

Current ML in olfaction builds on a research program with a straightforward question: What are the chemical features responsible for an odorant’s particular quality? By extension, this question is asserted to mean: What chemical features of an odorant allow for predictions about its olfactory quality? We think this line of inquiry is *deceptively* straightforward. Lloyd’s (2015) “Logic of Research Questions” (LRQ) will help us clarifying why this approach hides a critical premise about the nature of olfaction that (i) ML-models cannot account for and (ii) undermine confidence in the empirical adequacy of the correlations mined by these models.

LRQ is a method to analyze the conceptual constraints that the specific framing of a research question carries by clarifying the possible ranges of answers, their evidential hierarchy, and their contrast with other ways of framing that question. Before modeling any phenomenon, “[t]he most important feature of [research] questions is that each question carries with it an appropriate class of possible answers unique to it, and distinct from other contrasting classes of answers” (Lloyd, 2015). For example, Lloyd illustrates, a common question for examining the causal history and characteristics of a trait in evolutionary theory is: “What is the function of this trait?” However, this question (advanced by adaptationists such as Mayr, among others) is framed in such a way that it already presumes that the trait under investigation has a function. An alternative way of asking about the nature of a trait would be: “Does this trait have a function?” The logical difference between these two questions is visible in the answers that can be given in response to these questions, including the possible hierarchy of answers which might more likely be the case and their standards of evidence required. A similar analysis can be applied to ML-models in olfaction. Their current application builds on a research question with a causal model of the stimulus that has been questioned by a couple of scientific studies recently.

The research question asked by Keller et al. (2017), employed also in other ML-studies (e.g., Koulakov et al., 2011; Kumar et al., 2015; Kepple and Koulakov, 2017), is:

**Q1 *“Which chemoinformatic features of molecules predict their sensory (olfactory) attributes?”***

Possible and responsive answers to this question are:

A: *This* specific correlation, which is a feature F1 that can be found in *all molecules* belonging to the same odor category.
A: *That* correlation, which is different from the first correlation, in that it is a feature that can be found in *most* molecules belonging to the same odor category, specifically those molecules that all also have this other feature F2 {i.e., F1 and F2}.
A: This *other* correlation, which is different from the first two correlations, in that it is a feature that can be found in *most* molecules belonging to the same odor category, specifically those molecules that also have this other feature F2 or F3 or F4 {i.e., forming a class of disjunctive sets such as: (F1 and F2) or (F1 and F3) or (F1 and F4)}.
(And so on.)

Any answer to this question Q1 will follow this logical schema. Similar to the study of adaptive traits in evolutionary theory, we already start with a hidden assumption: that structural similarities of chemicals with the same odor account for the causal feature encoding the sensory information in question. But, as the mantra goes, correlation is not causation. Plus, this assumption is not theory-free. What is missing to interpret these similarities as causal requires a model of the mechanism with which these structural features are picked up by the system. Only against the backdrop of causal insight into the conditions of the biological system can we justify interpreting these *correlations* (between the microstructure of molecules and their odors *via* qualitative descriptions) *as causal.* Consequently, what kind of question must we ask if we want to target such causal features with our model? This points to the study of receptor-response causality.

Recent work (Poivet et al., 2016, 2018) successfully proposed an alternative in the study of the molecular basis of odor. This alternative concerns a causal model of the chemical stimulus in contrast to ML-studies. Moreover, this alternative relies less on an analysis of chemoinformatic properties of molecules. Instead, it centers “the biological responses of olfactory sensory neurons [to the stimulus]” (Poivet et al., 2018). Compared are not structural features of chemicals in isolation, but the responses of the receptors *to* chemicals, to identify the *causally responsive* features these chemicals may share in the living organism.

This alternative question reads:

**Q2 *“Which chemoinformatic properties are causally responsible in the detection of odorants by the (biological) system?”***

Possible and responsive answers to this question are:

A: *This* correlation, which is a feature F1 of molecules of the same odor category *where F1 is present in all receptors responding to these molecules*.
A: *That* correlation, which is different from the first correlation, in that it is a feature that can be found in molecules belonging to the same odor category *where F1 is present in most receptors responding to these molecules*, specifically those molecules where the same receptors also responded to feature F2 of these molecules {i.e., R (F1 and F2)}, [where R denotes “biological (or neuronal) response”].
A: This *other* correlation, which is different from the first two correlations, in that it is a feature that can be found in molecules belonging to the same odor category *where F1 is present in most receptors responding to these molecules*, specifically those molecules where the same receptors also responded to this other feature F2 or F3 or F4 {i.e., forming a class of disjunctive sets such as: R (F1 and F2) or R (F1 and F3) or R (F1 and F4)}.
(And so on.)

The two research questions, Q1 and Q2, are *logically different*. They contain different possibilities as their responsive answers. Yet, many members of the olfactory community prioritize chemistry-based over receptor-based models of the stimulus because they do not consider these two options *empirically different.*

For example, Mainland noted on the neglect of receptor-based models (Barwich, 2020a, 177): “We don’t need to know what the receptors are doing to figure out how to map structure to percept. The current [machine learning] models are basically doing that. (…). It can be a black box.” Keller, lead of the 2017 ML-study, agreed (ibid.): “You could predict from the physiochemical features what receptors it activates, and then you could predict from what receptors are activated what the perceived odor is. You just cut out that middleman and move over to black box off the receptors.” Similarly, Gerkin (ibid.), who developed a winning algorithm in Keller et al.’s (2017) study, responded: “We already know these receptors. We know about how many receptors there are. We broadly know how some of them are tuned, (…). But my point is that you can throw all that in the garbage. You can develop a theory of olfactory perception without knowing any of that. My hypothesis is that you can use psychophysics and make measurements to make strong predictions about the grand perceptual space, what the shape of the space is, and how stimuli mix in that space.” (Mainland and Gerkin may have changed their views since, see section “Biology makes scents of chemistry: receptor-response models of the stimulus”).

We object to this asserted parity of stimulus models based in chemistry and in biology. To clarify our reasons, we shall compare the design and results of Keller et al.’s (2017) ML-study with the wet-lab receptor research by Poivet et al. (2016, 2018).

## Analysis: Chemistry-based versus receptor-response models

The chemical concept of the olfactory stimulus originated in the late nineteenth to the mid-twentieth centuries. Key was the synthesis of coumarin in 1868 and vanillin in 1874 (Ohloff et al., 2012). The synthesis of odor compounds fueled a significant ontological shift: odors were disconnected from visible origins, such as plants and animal fats, and now belonged to invisible molecular causes of chemical features. How could mere chemical properties give rise to the rich mental imagery and perceptual qualities linked to odors in the human mind? (Barwich, 2020a, Ch. 1).

Biological notions of sensory perception did not have much traction until later in the twentieth century. Molecular biology, genetics, and associated disciplines were just in their infancy (Kay, 2000). Besides, the general concept of cell-surface receptors remained highly speculative and even contested until the 1980s (Barwich and Bschir, 2017). Consequently, chemical models of the stimulus were used to model hypothetical receptor sites in olfaction (Amoore, 1970), not vice versa.

The breakthrough transforming olfaction occurred in 1991 with Buck and Axel’s (1991) discovery of the odor receptor genes and their identification as GPCRs (Firestein, 2005; Firestein et al., 2014; Barwich, 2020a,b, 2021). However, receptor-research stalled for almost two decades. This lack of progress was methodological. Standard techniques such as heterologous expression (the expression of receptor genes in other tissues for functional studies) were successfully applied to the olfactory system only in the last decade (Dey et al., 2011; Mainland et al., 2015; Matsunami, 2016).

**FIGURE 2:**
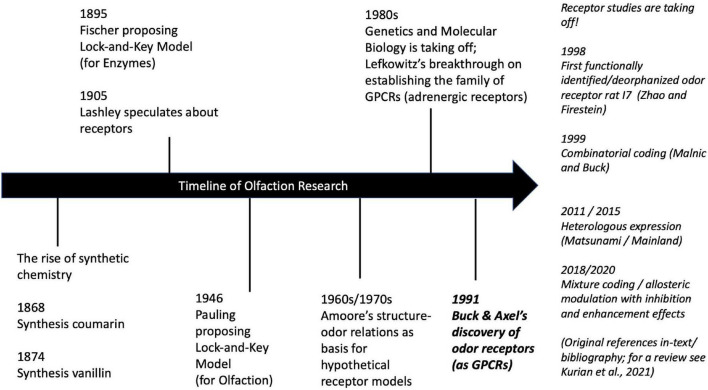
Historical timeline of selected key events in olfaction research (with focus on receptor biology). For details on the history of olfaction see Barwich (2020a, Ch. 1; biology-centered); and Ohloff et al. (2012, Ch. 1; chemistry-centered). For details on the history of genetics see Kay (2000) and GPCRs see Barwich and Bschir (2017). For a review of current research on odor coding mechanisms see Kurian et al. (2021).

This historical background (Figure 2) explains why chemical stimulus models dominated olfactory science. While the biology was long inaccessible to experimental manipulation, the chemical stimulus was available and controllable. Now that research on the receptors has considerably advanced, it is time to revisit the notion of the stimulus and models of its causal structure accordingly.

Current stimulus modeling follows the same trope: inference directly from the structure of molecules to sensory quality. Missing in this set-up is the biological interface of the system. Biology is consulted chiefly to explain prominent *irregularities* in chemically defined SORs (Barwich, 2015, 2018). But such apparent irregularities need not constitute exceptions. On the contrary, we contend that “irregularities” constitute “irregularities” only if we work with the wrong causal model. Researchers who use chemistry-based stimulus models are overlooking what they are building into their answers, omitting what *would* be truly explanatory: biology.

To clarify how insights from receptor biology inevitably alters our understanding of stimulus causality, we first outline how ML is used in olfaction to analyze its logical foundation and limits.

### From molecules to percept: current computational modeling of stimulus chemistry

Keller et al.’s (2017) study represents the most successful ML-account in olfaction to date. Its paradigmatic status resides less in its results: at 0.3, the correlation was not sufficiently high. What distinguishes this study from others of its kind is its experimental design.

Unlike its predecessors, Keller et al. (2017) used a new and extensive psychophysical dataset by Keller and Vosshall (2016), where 55 participants smelled and rated a (perceptually and structurally) diverse set of 476 molecules with 19 semantic descriptors (pulled from Dravnieks’ “*Atlas”*), in addition to scaling odor intensity and pleasantness. Prior studies of SORs, such as Koulakov et al. (2011), built exclusively on Dravnieks (1985) *The Atlas of Odor Character Profiles*, without consulting any psychophysical or experimental data. Notably, Dravnieks’ *Atlas* is a *lexical* compendium listing and scaling ∼146 verbal descriptors for odorants. This compendium was not intended to provide structure-odor regularities but designed for communicative purposes aiding a standardized description of odors in commercial application contexts. In other words, it is a trade lexicon, not a psychological research database. Keller and Vosshall (2016) cautioned about its use, as the “problem with verbal descriptors is that they are culturally biased. (…). Even if these descriptors were updated to be current and relevant across different nationalities and cultures, it is unlikely that semantic descriptors will ever cover the entire olfactory perceptual space.”

Additionally, what catches the eye in Keller et al. (2017) is its crowd-sourcing basis using DREAM challenges: an online platform for community-based research.

**FIGURE 3:**
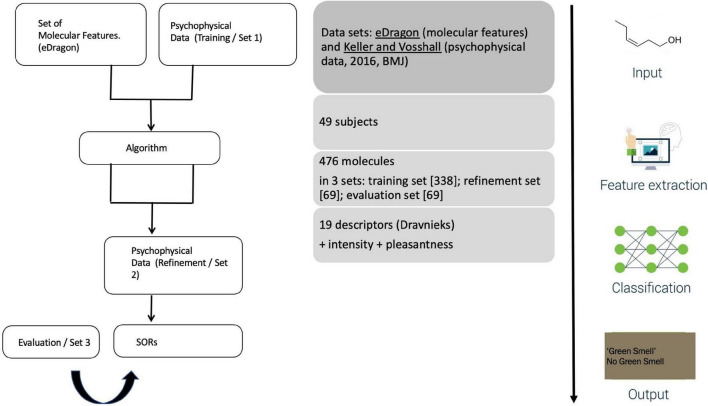
Keller et al.’s (2017) experimental design.

The study’s setup is straightforward (Figure 3). A public call with DREAM Challenges asked researchers to develop an algorithm accounting for two datasets: on the one hand, participants received a list of chemicals and, on the other hand, the results of the 2016 psychophysics study. This training set involved 338 molecules rated by 49 of the 2016 participants. ML-modelers were given an additional, smaller set (of 69 molecules from the 2016 study) to allow them to adjust their algorithms before submitting a final version. These submissions were evaluated with the remaining 69 molecules from the 2016 study.

How predictive were these ML-models, really? Keller et al. (2017) reported on two winning algorithms, not published with the paper and submitted by Guan (not an olfactory researcher) and Gerkin. Their winning algorithms gave structural predictions for 8 out of 19 descriptors with highly variable accuracy. Their findings, evaluating individual and population perception, presented the averaged (of all and only the best models) and individual model success in correlating molecular features with sensory attributes. For example, for individual perception, odor intensity was predicted with success rates of ∼0.8 (best individual algorithm), 0.5x (average of best models), and 0.4x (average of all models). Regarding odor quality, top predictions involved: garlic (best: 0.7; best average: 0.4x; average: 0.3), sweet (best: 0.6; best average: 0.4x; average: 0.3), and fruit (best: 0.6; best average: 0.2x; average: 0.2). Toward the lower end were: urinous (best: 0.6; best average: 0.4x; average: 0.3), wood (best: 0.5x; best average: > 0.1; average: > 0.1), and acid (best: 0.3x; best average: > 0.1; average: > 0.1). A comparison with population perception showed similar results with respect to best and lowest predicted sensory attributes of odorants.

What were the success criteria? Algorithms were evaluated by how well their identified set of features fit 1 of overall 19 labels (from which 8 of 19 were predicted), making a 0.3 correlation coefficient sound less exemplary. Besides, it seems spurious that DREAM challenges feature no shared conditions regarding the success of their miscellaneous contests, risking methodological arbitrariness among their ML competitions.

Criticism directed at the 2017 study involves five substantial concerns:^3^

First is the weakness of the *internal logic of the **descriptors*** and their selection:

Some are quite specific (garlic), other very broad (spices), and still others are ambiguous (chemical). What are we to make of “bakery” as a smell? Is it yeasty like baking bread? Is it the smell of fresh cinnamon buns? (…) The problem here is that *words that are useful in an olfactory lexicon occur at different levels of cognitive categorization* (Gilbert, 2017; added emphasis).

Linguistic descriptors are not representations *of perceptions*, and verbal descriptors do not directly account for sensory features. Without a psychophysical theory, the perceptual space and its characteristics remains opaque. In this context, the use of a linear set of descriptors in this and similar studies contains several misconceptions. Most stimuli carry ambiguous odors such that one odorant (i) contains multiple qualitative notes and (ii) can fall under various semantic categories (Barwich, 2020a). Ethyl citronellyl oxalate, for example, is musky *and* fruity. And the odor of butyric acid can fall under the label vomity and cheesy. ML-studies gloss over the inherent ambiguity of semantic tags in odor perception. Moreover, if ML algorithms can sieve through thousands of molecular parameters, should they not also be able to build an associated non-linear model of sensory concepts—addressing a hierarchical classification of sensory descriptors? (Say, “orange” and “lemon” falling under “citrus” which is “fruity.”) Overall, the adequacy of verbal descriptors for the causal analysis *of the stimulus* is questionable: “In 1988, Chastrette et al. (1988) (1) studied a collection of 2,500 odor descriptions (2) and concluded that only 3% of the descriptors led to a fruitful odor-structure relationship” (Poivet et al., 2018).

The second criticism, which links to the above, concerns the tacit *assumption that SORs are **innate***. However, human odor perception, including its verbal labeling, is heavily influenced by experience, context, and culture (Herz and von Clef, 2001; Majid and Burenhult, 2014; Barwich, 2020a). Notably, even in mice we find that the responses to seemingly “innate” odors are affected by context and can be altered (Qiu et al., 2021a,b).

Third is the *limited **generalizability*** of tested substances and descriptors:

if one wants to predict what molecules might smell of sandalwood or citrus, one would have to *retest* all 476 molecules on another forty-nine sensory panelists using the new list of descriptors, then rerun the computer models on the new data set (Gilbert, 2017; added emphasis).

More than prediction in the strong sense, these algorithms performed a classificatory function. Compared with the periodic table of elements, facilitating predictions about the existence of hitherto unknown elements (Scerri, 2019), the “soft predictive power” of these ML-algorithms remained confined to existing data. To be genuinely predictive, these algorithms need to fulfill stronger philosophical criteria (Barrett and Stanford, 2004). Predictions involve the discovery of ‘new’ data, and new means that the findings of a model must not be entailed by existing data. The 2017 finding that molecules with sulfur atoms tend to smell sulfurous, therefore, is not a new discovery given existing knowledge. Instead, it retracks and confirms correlations in existing data. Further, predictions are often formulated as implications or entailments of a scientific theory or model. But there is no model explicated on the basis of these algorithms. Additionally, only a couple of findings from these algorithms were translated into predictive rules (*if* property P1 *then* effect E1)—or *quasi*-predictive, as they expressed approximations. For example: “[t]he presence of sulfur atoms makes things *more likely* to smell burnt or garlicky. Bigger molecules are *more likely* to smell pleasant” (Yong, 2016; added emphasis).

Exceptions to these algorithmic (chemical) correspondence rules may be seen to exemplify the known issue of *irregularities* in structure-odor-modeling, and are presented almost as a built-in feature of the olfactory system (i.e., it’s not a regular system anyway because biology messes with ideas of lawful regularity). But what if these “irregularities” are consequences of a false modeling premise, given that there is a complete biological model missing in those explanations? We suggest such irregularities appear only if you start with the principles of chemistry. They may not constitute irregularities and cease to be exceptions in biological models of the stimulus. Indeed, this connects with the following criticism.

Fourth are the ***biological variables*** that causally account for known perceptual variations in response to the chemical stimulus.

One example here is the *polymorphism* of cell receptors, where a single interface (odor receptor) can respond to numerous variables (odorants) and produce multiple different response types (odors). An example are mixed responses to androstenone, which individuals perceive quite differently as urinous, sweaty, woody, or fruity (Wysocki and Beauchamp, 1984). Such high perceptual variation has been linked to receptor polymorphism (Keller et al., 2007).

Another example is *receptor tuning* which does not correspond neatly with sets of chemical similarities (studied with glomeruli: Soucy et al., 2009; Ma et al., 2012). Widespread variation in tuning ranges appears to be an intrinsic property of mammalian odor receptors (Kepchia et al., 2017). A critical consequence of this non-overlap of chemical properties in receptor-tuning profiles is that activation patterns in the olfactory bulb do not allow for a chemotopic mapping of chemical properties with glomeruli (Barwich, 2020a, Ch. 7), having significant implications for models of central processing and perception.

Moreover, odorants do not reach the receptors unmediated but interact with enzymes in the olfactory mucosa. These *perireceptor events* can alter the odorant’s composition and thus result in altered compounds with a different response (Nagashima and Touhara, 2010; Heydel et al., 2013; Asakawa et al., 2017).

Further, once we examine mixture perception, *odorants exhibit multiple functional roles and causal profiles* in receptor interactions. For example (Kurian et al., 2021), odorants are “acting on receptors as agonists, antagonists, inverse agonists, partial agonists, and even have a synergistic effect (Reddy et al., 2018; Ikegami et al., 2020; Inagaki et al., 2020; McClintock et al., 2020; Pfister et al., 2020; Xu et al., 2020; Zak et al., 2020).”

Finally, a fifth criticism is methodological and concerns the ***linearity*** in current ML-models of SORs. The non-linearity of receptor responses to odorants in contrast with the linear modeling in current computational approaches to SORs may account (at least partially) for the latter’s present inadequacies.

Overall, and to tie this section together, current computational approaches for the prediction of SORs all build on the starting assumption of a *direct correspondence* between odorants (the stimulus causing the smell, characterized by organic chemistry) and psychodescriptive labels. Such models only study chemical properties in their correlations to assigned verbal labels.

ML-models of SORs give a descriptive account of chemical similarities from the specific perspective of organic chemistry. But they cannot provide justification for the belief that these features are causally relevant. Thus, correlations expressed by these models provide *leads* for a hypothesis and not an explanation of why and how these features are causally active. With experiments following the logic of research question Q1 (section “Deep nose and the logic of research questions: theory in machine learning studies in olfaction”), we have no way of knowing whether their findings account for *how the system processes and codes smells*, not even at the periphery. In turn, we cannot claim to have arrived at a model connecting the perceived qualities to molecules *biologically*. However, that is what determines the causality of the features of the stimulus.

We think the omission of biology in current ML-models in olfaction is profoundly mistaken. Moreover, we caution about the logic of the research question Q1 implicit in these models: it does not constitute a benign research program because it works on misleading assumptions about what is truly causal and explanatory.

In our view, the absence of causal considerations and focus on semantic labels is problematic, no matter how predictive of SORs similarly designed ML-models may be in the future. Chemoinformatic properties of (i) single molecules (ii) in isolation have little bearing on understanding the real-world mechanisms of odor perception. Humans smell plumes of multiple compounds where the sensory system can distinguish between target and background odors. Notably, the same “odor object” (meaning the assignment of semantic object labels to a sensory quality, e.g., rose) can refer to mixtures of *varying molecular composition*. – Meanwhile, receptor-centered emphasis in this paper must not lead to the misleading impression that models of perception can be inferred directly from receptor-response models instead of SORs. Central to odor coding are delicate circuitry and developmental processes in the olfactory bulb (Cleland and Sethupathy, 2006; Maresh et al., 2008) and recent discoveries such as representational drift in piriform (Schoonover et al., 2021), which further question SORs models (Barwich, 2020a).

Historically, it made a great deal of sense that chemical notions of the stimulus took over olfactory research throughout the twentieth century. We question whether it continues to be an apt modeling strategy today. Because if the research problem is misconceived, it matters little if you are using a new tool to accelerate its application.

### Biology makes scents of chemistry: Receptor-response models of the stimulus

Is there sufficient reason to think that knowledge of receptors and their behavior may change our understanding of odor coding? Indeed, there is (Barwich, 2020a,^2021^, forthcoming). A chief reason for consulting the biology of the system in models and explanations of the olfactory stimulus concerns the fact that, as of recently, we could not even predict whether a molecule has a smell at all:

it is nearly impossible to predict whether a given molecule will be odorous and what its odor quality might be from the chemical structure alone. Although all odor molecules are typically organic compounds of low molecular weight, they may be aliphatic or aromatic, may be saturated or unsaturated, and may have any of several polar functional groups. However, there are many molecules that conform to those characteristics, which are nonetheless odorless, to humans and other animals (Poivet et al., 2018).

In response to this challenge, Mayhew et al. (2022; added emphasis) reported that their computational model identified features that might help explain and predict which molecules are odorous: “We found that molecules with sufficient volatility and hydrophobicity are generally odorous, which suggests that *reaching olfactory receptors is the dominant barrier* for prospective olfactory stimuli.” The causally significant stimulus properties were features involved in the various biological processes involved in binding the stimulus with the receptors. More simply: biology determined the modeling of chemistry (Gerkin and Mainland were involved in this study).

This example (Mayhew et al., 2022) illustrates why the issue in our view is not the general application of computational tools such as ML in olfaction. As mentioned earlier, our critique targets the *adequacy of the central assumptions hidden in the currently predominant use* of machine learning models in olfaction. Once its assumptions are made explicit, using LRQ as in this article, ML offers a tremendous technique to derive possible leads that act as a heuristic for future research into correlations that may be causal in nature. Testing of these correlations as causal, however, requires (i) empirical follow-ups and experiments, and (ii) a model of a causal mechanism in which such structural correlations provide causal explanations (Barwich, 2021). The results of ML thus aid scientific hypothesis generation and modeling but do not constitute evidence independent of theory and wet-lab experiment. ML thus conceived carries great potential to highlight certain chemical features to be tested against the receptors.

Still, skeptics may raise the question: why should we *start* with receptor responses in modeling the chemical stimulus of smell? This question carries appeal with the case of Mayhew et al. (2022), suggesting that we could arrive at features relevant to receptor responses. We consider this optimism misguided, though. First, it presents a mistake in terms of quantificational logic: it makes an illegitimate inductive inference by claiming an “all” quantifier with the case of a “some” quantifier. Second, beyond *logical fallacy*, there is sufficient *empirical evidence* in support of our argument.

We shall highlight two sibling-studies emphasizing biological function over chemical form: Poivet et al. (2016, 2018) analyzed receptor responses to identify physico-chemical features critical to (i) how the receptors bind an odorant and (ii) which odorants are perceived as more similar than others. Poivet et al. (2018), was set up explicitly in corrective contrast with Keller et al. (2017):

One approach recently used to solve [the problem of odor coding] was to apply machine learning strategies to a large set of odors and human classifiers in an attempt to find common and unique chemical features that would predict a chemical’s odor. We use an alternative method that relies more on the biological responses of olfactory sensory neurons and then applies the principles of medicinal chemistry, a technique widely used in drug discovery.

**FIGURE 4:**
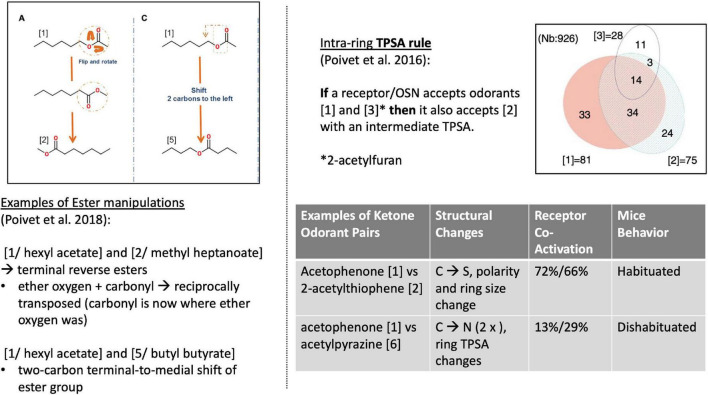
Left: Structural comparison of the 3 esters (Poivet et al., 2018). Right [top: TPSA rule (Poivet et al., 2016)]; bottom: 2 examples of receptor co-activation in ketone pairs (Poivet et al., 2018).

Poivet et al. first tested receptor responses to 6 ketones in 2016, followed by a study of receptor responses to 5 esters and 1 ketone in 2018, *via* the activity of dissociated olfactory cells with calcium imaging. (Olfactory neurons typically express one receptor gene (Chess et al., 1994), and are widely used as experimental substitutes for receptor binding studies in olfaction.) All test odorants were chosen considering their structural composition: each odorant had one slight difference to a reference odorant in the study (Figure 4, left). For example: “In the case of the esters used here, the position of the ether oxygen and the lengths of the carbon chain on either side of the ether oxygen or the carbonyl group were varied, and we investigated the reverse esters of each compound—esters in which the ether and carbonyl oxygens are transposed compared to the original compound” (Poivet et al., 2018).

**FIGURE 5:**
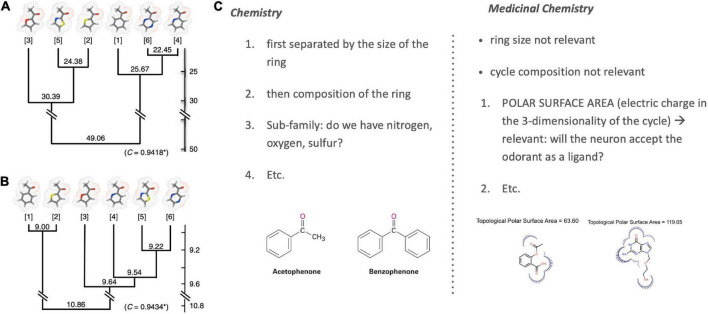
Classification of odorant similarity according to analytic and medicinal chemistry. Illustrated is the difference between the classification of the chemical similarity of ketones (left) according to **(A)** the principles of analytic chemistry and **(B)** medicinal chemistry and receptor behavior [image from Poivet et al. (2016); OSN stands for Olfactory Sensory Neurons]. Differences are especially visible when comparing the closest similarity pairs in **(A)** analytic chemistry {4; 6} and **(B)** medicinal chemistry {1; 2}. These differences are grounded in varying ordering criteria of chemical similarity between chemistry and medicinal chemistry [example right; **(C)**] [Note: Sample molecules in panel **(C)** are not ketones but chosen merely for an illustration of the selection criteria].

Poivet et al. compared the number of receptors co-activated by these odorants before systematically checking for the salient physico-chemical features in receptor-coactivating odorants (Figure 4, right). This receptor-based classification of odorant similarity was contrasted with a similarity tree derived based on organic chemistry. Comparing these two similarity dendrograms (Figure 5, left), they demonstrated that odorant classifications yielded substantially different similarity trees depending on whether one arranges odorants with the principles of analytic chemistry or receptor responses in medicinal chemistry.

This work revealed two key findings. First, it showed that the *categorization* of the same class of odorants (here: ketones and esters) *diverged substantially* between the two causal strategies (stimulus-centered versus receptor-centered). Second, receptor-based modeling highlighted fundamentally *different physicochemical features as the causally relevant ones* (Figure 5, right). Possibly the most surprising and striking discovery was that a key causal features of ketones (correlated with odorant co-detection by receptors) was the Topological Polar Surface Area (TPSA; Figure 4), a feature playing little role in chemistry-based classifications.

But what about *behavior*? An advantage of Keller et al. (2017) was their inclusion of a new human psychophysical dataset. Poivet et al.’s (2016, 2018) studies used disassociated cells from genetically engineered mice. Could these mice also distinguish these odors to different degrees? In response, Poivet et al. (2016) undertook (dis)habituation tests with mice: “Habituation is defined by a progressive decrease in olfactory investigation toward repeated presentation of the same odor stimulus. Dishabituation is defined by reinstatement of olfactory investigation when a novel odor is presented.” Indeed, mice demonstrated (i) reciprocal habituation to odorants that co-activated the same receptors and (ii) dishabituation to odors dissimilar according to receptor responses (i.e., less co-activation), illustrating that OSN response patterns are a better predictor of odor behavior than chemistry.

Yet what about humans? Poivet et al. (2018) tested the different esters and asked a small panel of participants to rank them. Results were interesting. On the one hand, humans behaved like the mice: they “perceive the medial esters [5] and [6] to be the most similar.” On the other hand, we find differences: “unlike mice, [participants] classified them as closer to [2] than to [3]. Humans also clustered [1] with [3] rather than [2].” Additionally, humans showed a higher variety in similarity responses than the mice. Contrary to intuition, this favors biology-centric modeling because the human olfactory system is genetically highly diverse, resulting in receptor-response differences that have been linked to perceptual variation (Mainland et al., 2014; Trimmer et al., 2019). In comparison, mice are genetically homogeneous model organisms (Ankeny and Leonelli, 2020), and would show less variation in behavioral responses to odorants.

Further, can receptor-based models arrive at structure-activity rules and, if so, how *predictive* are such rules? The appeal of Keller et al. (2017) was their claim that ML-models may lead to SORs with predictive value. Yet, we also find receptor-based response rules with predictive value for odorant similarity.

Poivet et al. (2016) elaborated on the possibility of deriving structure-activity rules from receptor co-activation. For example, they posited the “TPSA-transferability rule” stating that: if a receptor (group) is co-activated by an odorant with a TPSA value [1] and an odorant with increased TPSA value [3], then this receptor (group) will also be activated by an odorant with an intermediate TPSA value [2]. They further tested whether this rule applied to ketones beyond the test panel. It did. Such findings constitute discoveries of *new* data not previously entailed in the datasets and model. In this context, one final blow to the lingering skeptic is that receptor responses markedly question our established chemical definitions of odorant classes:

we find that there is a strong relation between esters and ketones that is not predicted by chemical analyses. This unexpected result led us to theorize that the critical feature of ester molecule discrimination is not the ester group but rather the position of the carbonyl group (Poivet et al., 2018).

Lastly, how experimentally *productive* and *expandable* is medicinal chemistry as a research program? Recently, Burton et al. (2022) tested activation in the bulb to 185 odorants and analyzed stimulus similarity patterns with medicinal chemistry. Instead of traditionally received chemical spaces, this study mapped “response spectra” of glomeruli according to receptor sensitivity and tuning. Sometimes old problems need not new tools but a new perspective.

Inevitably, biology-based studies are comparatively small in scope and more localized in their probing of causal details when compared with big ML-studies. A better understanding of odor coding and causality must build on an approach that *iteratively combines* these tools, ML-studies with receptor-based modeling, to test the rules at which they, respectively, arrive. Hence, we suggest adding the utility of computational tools to question Q2 and its research program. Rather than denying the obvious potential of the tool, we simply advocate for a different causal grounding of it to discover more about the molecular basis of olfaction.

Meanwhile, first results of computational approaches to receptor-response models of the stimulus are emerging. But this research does not provide transparent answers by magic. While receptor-based ML-approaches show notably higher correlation results (sometimes up to ∼70%), some of these studies also delivered non-overlapping clusters of chemical features in direct comparison. The reasons behind such divergence appear methodological. As proof-of-principle, Haddad et al. (2008a) tuned a bioelectronic nose to the receptive range of the de-orphanized rat receptor I7 (Zhao et al., 1998; Araneda et al., 2000), thus extrapolating chemical features based on the response activity of a single receptor. They further applied this set-up to 24 drosophila receptors. Meanwhile, Haddad et al. (2008b) began with a multidimensional metric re-applied *via* meta-analysis to 9 datasets from previous studies. In contrast, Saito et al. (2009) heterologously expressed 464 receptors, and functionally tested 93 odorants yielding an agonistic profile for 52 mouse and 10 human receptors—extrapolating chemical features based on the response profiles of multiple and genetically diverse receptors. While Saito et al. began with data production from wet-lab research, Haddad et al. began with computationally derived parameters compared against existing wet-lab datasets. Thus, meaningful metrics comparing and measuring the success of such hybrid modeling requires closer analysis concerning (i) their varying methodological and (ii) assumed biological conditions.

Overall, receptor-based models provide significant empirical reasons to caution against the notion of taking ML-models as revealing causal features without a systems-theoretic background. From this complementary perspective, receptor-based studies yield ontological priority and do *not* take a backseat since (i) chemical similarity modeled in isolation cannot account for receptor-coactivation and (ii) datasets with chemical diversity need not reflect biological response diversity. With this approach, biological-response accounts are not used to explain anomalies, as in previous chemistry-based accounts, and, instead, are the very basis of the causal theory used in explanation.

## Conclusion

This article analyzed the patterns of inference and explanation in ML-models applied to olfaction. We saw that the logic of research questions in chemistry-based models of the stimulus in prominent computational approaches markedly differs from biology-based modeling in wet-lab research (section “Deep nose and the logic of research questions: theory in machine learning studies in olfaction”). Specifically, we assessed two sets of research questions, each with distinct possible and responsive answers: one, Q1, is mostly chemical and building on a research tradition from the twentieth-century before the olfactory receptor discovery; and the other, Q2, which has been coming into experimental focus only very recently, is mostly biological. We argued that a biology-centered approach with its associated research question Q2 ultimately yields the answers that we seek in understanding the coding principles of the olfactory system. In addition to logical differences, we evaluated the available empirical evidence of the leading studies representative of each approach (section “Analysis: chemistry-based versus receptor-response models”). In this context, we find that receptor-response studies widen our possibilities in explaining odor coding concerning definitions of odorant similarity and their connections with perceptual behavior.

Our general critique from the argument in this article is that if we continue using the chemistry-based approach, then we also continue building models of olfaction on a *fundamentally mistaken notion of odor coding as a sensory process*. Theorizing about perception routinely presents the following story: the brain extrapolates information *via* an efficient feature extraction process by filtering general information from contingent scenarios (Marr, 1982). But what are the truly significant bits? And how does the sensory system represent those: is it according to the currently dominant chemistry-based or a biology-based model of smell? This line of question is considered irrelevant for many ML-approaches to smell (Wiltschko, 2019; section “Deep nose and the logic of research questions: theory in machine learning studies in olfaction”). But they are not, as we just showed: we get different answers, depending on which research question and theoretical basis we use.

Ultimately, we suggest that a combinatorial approach of biologically informed machine learning appears to present the most fruitful program for future research. We therefore hope the future brings greater attention, science-journalism appeal, and funding to research into the biology of the system. Unfortunately, receptor-response studies are underfunded and far less easy to “just throw out there” in comparison with ML-approaches drawing on existing datasets. Meanwhile, obstacles for the successful acquisition of receptor datasets remain. For example, (1) we do not have the crystal structure of the mammalian odor receptors; (2) not all receptors are de-orphanized (Peterlin et al., 2014), meaning their ligand binding range remains unknown. These gaps in our present knowledge carry consequences for causal models of receptor biology and stimulus interactions. Without crystal structures, detailed insight and comparisons of the molecular architecture of receptors remain limited. Additionally, functional understanding of the range and variety of odorants to which a receptor can bind requires systematic de-orphanization. As long as localized receptor studies are sidelined in favor of quick and dirty ML-studies, we won’t be able to evaluate which chemical features are *functionally* (that is, biologically and operationally) involved in receptor co-activation—further allowing for inferences to functional rules to be used in ML-studies. That said, critical breakthroughs in research on odor receptor function and coding mechanisms—often with the introduction of new experimental tools—were seen to emerge in the past couple of years (Kurian et al., 2021).

In the end, to make machine learning modeling truly informative, more wet-lab and bench-work (meaning tissue studies on the receptors in empirical laboratory settings) is needed, as AI cannot give you that kind of data.

## Author contributions

A-SB analyzed the modeling strategies in olfaction. EL developed the philosophical framework for analysis. A-SB and EL wrote the manuscript. Both authors contributed to the article and approved the submitted version.

## Footnotes

1 For related criticisms and arguments concerning the task-specificity and theory-ladenness of big data and computational techniques, including deep learning, see Lloyd et al. (forthcoming) for climate modeling and Dupre (2021) for linguistics.

2 The fragrance industry, a > 25-billion-dollar enterprise, has accumulated large proprietary databases in their research and development departments involving odorant design and consumer research. Thus, one may suspect these companies have been conducting less publicized and equally (un)productive attempts at SORs ML-modeling.

3 We are *immensely* grateful to all three reviewers for their extraordinarily helpful suggestions in systematizing and expanding these points of criticism.
